# *Begonia myanmarica* (Begoniaceae), a new species from Myanmar, and molecular phylogenetics of *Begonia* sect. *Monopteron*

**DOI:** 10.1186/s40529-017-0175-9

**Published:** 2017-04-07

**Authors:** Yu-Hsin Tseng, Young-Dong Kim, Ching-I Peng, Khin Myo Htwe, Seong-Hyun Cho, Yoshiko Kono, Kuo-Fang Chung

**Affiliations:** 1 Research Museum and Herbarium (HAST), Biodiversity Research Center, Academia Sinica, Taipei, 11542 Taiwan; 2 Department of Life Science, Hallym University, Chuncheon, Gangwon 24252 Republic of Korea; 3 Popa Mountain Park, Nature and Wildlife Conservation Division, Ministry of Natural Resources and Environmental Conservation, Kyaukpadaung Township, Mandalay Region Myanmar; 4 International Biological Material Research Center, Korea Research Institute of Bioscience and Biotechnology, Daejeon, 34141 Republic of Korea

**Keywords:** *Begonia griffithiana*, *Begonia nepalensis*, Chromosome, Morphology

## Abstract

**Background:**

A new species, *Begonia myanmarica*, was discovered from Myanmar and herein documented. Characterized by a single developed wing in the ovary/fruit, this species would be assigned to sect. *Monopteron* (sensu Doorenbos et al. in The sections of *Begonia* including descriptions, keys and species lists: studies in Begoniaceae VI. Wageningen Agricultural University, Wageningen, 1998) that is known by *B. griffithiana* and *B. nepalensis* from the Himalaya. To confirm its sectional assignment, we conducted morphological, phylogenetic and cytological studies.

**Results:**

Morphological observations indicated that *B. myanmarica* was distinguishable from the two known species of sect. *Monopteron* by the leaf shape and size, 1-locular ovary, parietal placentation and chromosome number. Molecular phylogenetic analysis using nrITS sequences showed that *B. myanmarica* was not allied with the clade of sect. *Monopteron*, though both were nested within sect. *Platycentrum*-sect. *Sphenanthera* clade.

**Conclusions:**

Studies of morphology, molecular phylogenetics and cytology support the recognition of the new species, *Begonia myanmarica*, which is fully described and illustrated. Our results also indicate that *B. myanmarica* is not closely related to species previously assigned to sect. *Monopteron*, suggesting that the fruit morphology of a single developed wing in the ovary/fruit characterizing sect. *Monopteron* is homoplasious.

## Background

*Begonia* L. (Begoniaceae), comprising more than 1800 species classified into 68 sections (Doorenbos et al. 1998; Hughes et al. 2015; Christenhusz and Byng 2016), is one of the largest genera of vascular plants. With more than 760 *Begonia* species in Asia, Doorenbos et al. (1998) recognized 18 sections [*Alicida* C.B. Clarke, *Apterobegonia* Warb., *Baryandra* A. DC., *Bracteibegonia* A. DC., *Coleocentrum* Irmscher, *Diploclinium* (Lindl.) A. DC., *Haagea* (Klotzsch) A. DC., *Heeringia* Irmscher, *Lauchea* (Klotzsch) A. DC., *Monophyllon* A. DC., *Monopteron* (A. DC.) Warb., *Parvibegonia* A. DC., *Petermannia* (Klotzsch) A. DC., *Platycentrum* (Klotzsch) A. DC., *Putzeysia* (Klotzsch) A. DC., *Reichenheimia* (Klotzsch) A. DC., *Ridleyella* Irmscher, and *Sphenanthera* (Hassk.) Warb.]. Thereafter, four additional Asian sections were proposed [*Leprosae* (T.C. Ku) Y.M. Shui, *Monolobium* T.C. Ku, *Pleiothece* T.C. Ku, and *Symbegonia* (Warb.) G. Forrest & Hollingsw.] (Ku 1999; Shui et al. 2002; Forrest and Hollingsworth 2003; Ku et al. 2007). These 22 Asian sections are highly unequal in species numbers: eight of the large sections (*Petermannia*, *Platycentrum*, *Diploclinium*, *Reichenheimia*, *Coleocentrum*, *Parvibegonia*, *Sphenanthera*, and *Symbegonia*) comprise 95% of Asian *Begonia* species and the rest 14 sections each with less than five species (Thomas 2010). Several molecular phylogenetic studies have demonstrated the paraphyly or polyphyly of these large sections, suggesting homoplasy of morphological characters used for current sectional delimitations (Tebbitt et al. 2006; Thomas et al. 2011; Chung et al. 2014). However, few studies have tested the monophyly of small Asian section of *Begonia* thus far [but see Rajbhandary (2010); Rubite (2010); Thomas (2010)].

Myanmar is botanically a most interesting country, but there have been no critical floristic surveys for nearly half a century. Thus far about 60 species of *Begonia* have been recorded from Myanmar (Hughes 2008; Tanaka and Hughes 2007; Tanaka and Hayami 2011; Peng et al. 2014b; Tanaka and Peng 2016). During the fieldwork in western Myanmar on 2 February 2012, the second author (YDK) collected an unknown *Begonia* with only one developed wing in ovary/fruit, which is the key character of *Begonia* sect. *Monopteron* sensu Doorenbos et al. (1998) first delimited by de Candolle (1864) as *Mezierea* sect. *Monopteron*. Presently, only two species, *B. griffithiana* Warb. and *B. nepalensis* Warb., are recognized in sect. *Monopteron* (de Candolle 1864; Doorenbos et al. 1998). *Begonia nepalensis*, the type species of sect. *Monopteron*, is native to Bhutan, Nepal and India (Fig. 5; Doorenbos et al. 1998; Hughes et al. 2015). Its chromosome number was reported to be 2*n* = 16 (Legro and Doorenbos 1971), with an uncertain chromosome count 2*n* = 28–42 by Sharma and Bhattacharyya (1961). *Begonia griffithiana*, occurring in Bhutan and India (Fig. 5), is characterized by lanceolate to oblong leaves with subcordate base. Chromosome number of *B. griffithiana* was documented as 2*n* = 22 (Doorenbos et al. 1998). Based on recent systematics and phylogenetics of *Begonia*, sect. *Monopteron* is nested within the *Platycentrum*-*Sphenanthera* clade (Rubite 2010; Thomas 2010; Rajbhandary et al. 2011; Leong 2017).

Although morphology of the 1-winged ovary/capsule of the undescribed *Begonia* should be assigned to sect. *Monopteron*, it differs from *B. griffithiana* and *B. nepalensis* significantly the leaf shape, leaf size and distribution. In this study, we described it as a new species. We also provide detailed morphological data and molecular phylogenetic analysis to elucidate the sectional assignment for this species.

## Methods

### Morphological observations

Rhizomes of *Begonia myanmarica* collected by YDK from Myanmar were cultivated in the experimental greenhouse of the Biodiversity Research Center, Academia Sinica, Taipei, Taiwan. Fully grown plants with flowers and fruits (*Peng 23565*, *23566*) were used for morphological observation. The two species of sect. *Monopteron*, *B. griffithiana* (*Peng 20851*) and *B. nepalensis* (*Peng 20854*), cultivated in the greenhouse were also studied as a comparison.

### Chromosome preparations

Root tips were obtained from cultivated materials from greenhouse of Academic Sinica. Somatic chromosome of the new species, *B. myanmarica* (*Peng 23566*), and two species of sect. *Monopteron*: *B. griffithiana* (*Peng 20851*) and *B. nepalensis* (*Peng 20854*), were examined using root tips following the methods by Peng et al. (2014a).

### Phylogenetic analyses

DNA sequences of the nuclear ribosomal internal transcribed spacer (nrITS) were used to evaluate the phylogenetic relationship among new species and the two species of sect. *Monopteron*. DNA extraction, PCR amplification and DNA sequencing followed Chung et al. (2014). To test the monophyly of sect. *Monopteron* and sectional assignment of new species, nrITS of 96 species used in Chung et al. (2014) were adopted for phylogenetic analysis (see Appendix for details). Alignment was conducted using MUSCLE implemented in MEGA5.2 (Tamura et al. 2011) and verified in Mesquite v3.03 (Maddison and Maddison 2015). Phylogenetic relationships were constructed by Bayesian Inference (BI) method. The best nucleotide substitution models were determined by Modeltest v2.7 (Posada and Crandall 1998). For BI analysis, the consensus topology was based on Markov chains algorithm implemented in MRBAYES 3.0b4 (Huelsenbeck and Ronquist 2001). Four chains of Markov chain Monte Carlo (MCMC) simulation were carried out for 1,500,000 generations each with trees sampled per 500 generations. The first 500 trees of sampled trees were discarded before the node probability was calculated (posterior probability: PP).

### Taxonomic treatment

**Fig. 1:**
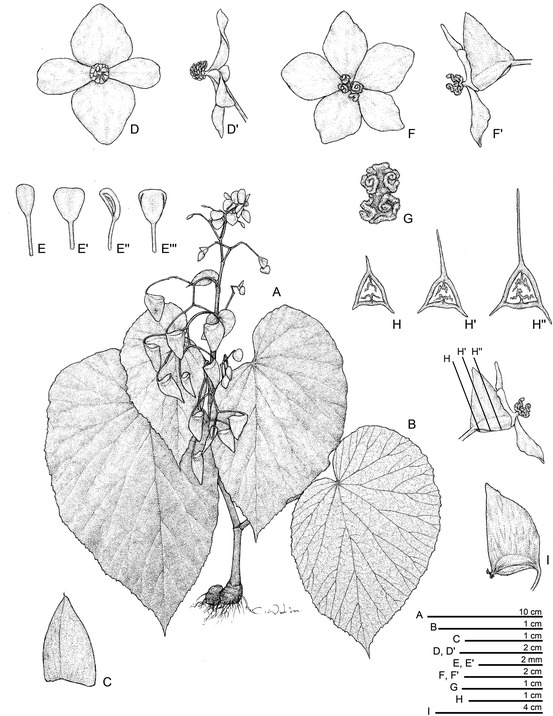
*Begonia myanmarica* C.-I Peng & Y. D. Kim. **A** Habit. **B** Leaf adaxial surface. **C** Stipule. **D** Male flower, face view, **D′** Male flower, side view. **E, E′, E″, E‴** Stamen. **F** Female flower, face view, **F′** Female flower, side view. **G** Style and stigma. **H, H′, H″, H‴** Cross section of ovary. **I** Capsule

**Fig. 2:**
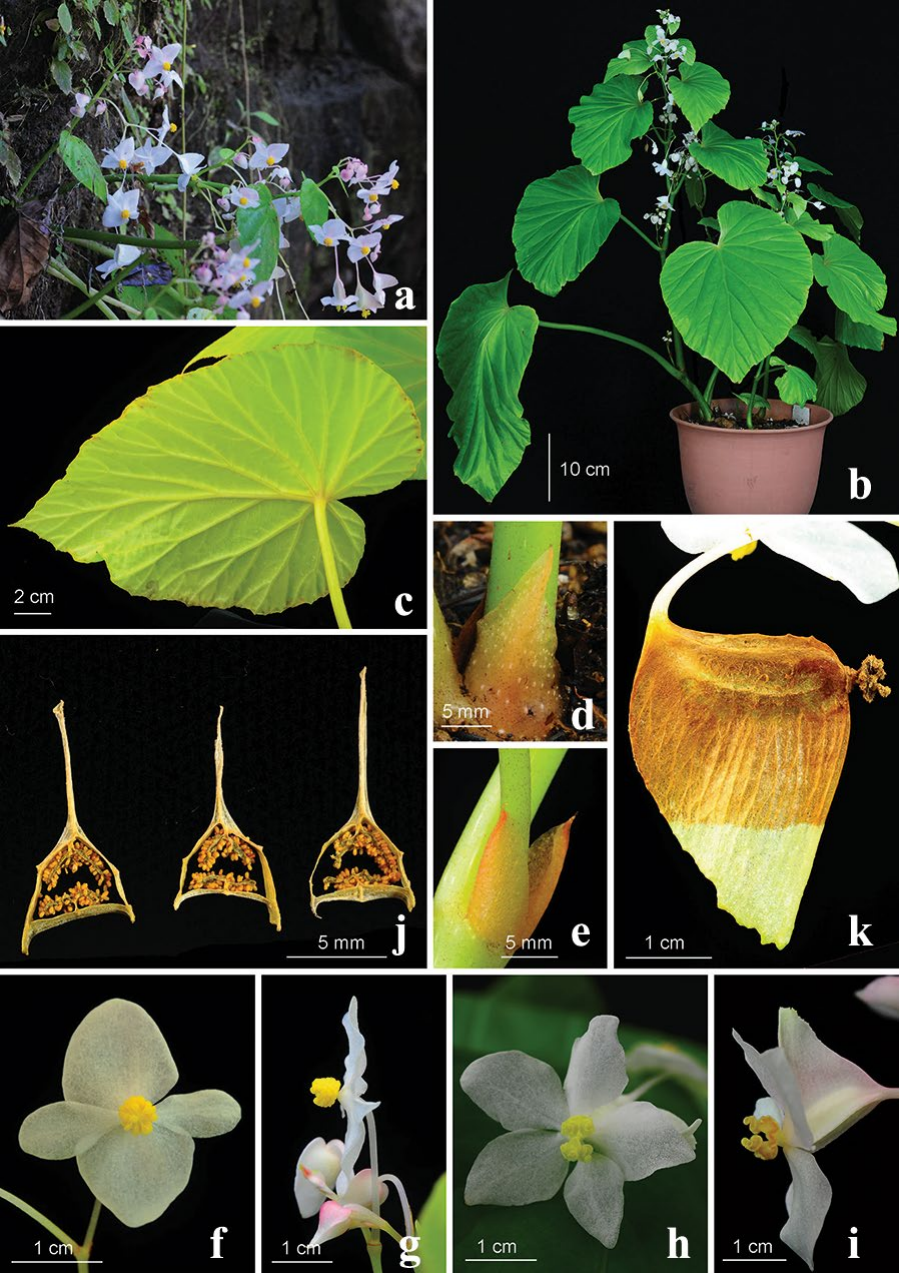
*Begonia myanmarica* C.-I Peng & Y. D. Kim. **a** Habit and habitat. **b** Cultivated plant at anthesis. **c** Leaf abaxial view. **d** Stipule. **e** Bract. **f** Male flower, face view. **g** Male flower, side view. **h** Female flower, face view. **i** Female flower, side view. **j** Cross section of ovaries. **k** Capsule

**Begonia myanmarica** C.-I Peng & Y. D. Kim, sp. *nov.* (Figs. 1, 2)

### Type

MYANMAR. Sagaing Region, Alangdaw Kathapa National Park, 22°18′ 47.7″ N, 94°28′32.7″ E, alt. 438 m, mixed deciduous forest, along the stream. Living collection made by *Seong*-*hyun Cho*, *Young*-*dong Kim*, *Yong*-*in Kim & Jeong*-*hun Lee MM*-*0611*, 2 Feb 2012; type specimens (with flowers and fruits) pressed from plants cultivated in the experimental greenhouse, Academia Sinica, Taipei, Taiwan, 20 Mar 2016, *Ching*-*I Peng* 23566 (holotype: RAF; isotypes: HAST, KB).

### Diagnosis

*Begonia myanmarica* is a unique species with an erect habit; large, ovate to broadly ovate leaves (ca. 20–40 cm long, 22–30 mm across); sole, much protruded wing in ovary/fruit; 1-locular ovary with parietal placentation and 2 placentae; and the somatic chromosomes are determined as 2*n* = 38.

Herbs monoecious, perennial. Rhizomes stout, 2–4 cm across, to 9 cm long; erect stem 50–90 cm tall, 1–2 cm thick, internodes 5–15 cm, glabrous. Stipules caducous, glabrous, triangular, apex aristate or apiculate, margin entire, 7–15 mm long, 6–15 mm wide. Leaves alternate, green with slightly paler veins; petiole 20–40 cm long, 2.2–3 cm across, glabrous; leaf blade fleshy, asymmetric, ovate to broadly ovate, 21–35 cm long, 15–28 cm wide, upper surface glabrous, underside slightly hairy on veins, base obliquely cordate, margin irregularly loosely serrulate or denticulate, apex acute or short acuminate; venation 7-or 8-palmate. Inflorescence mainly terminal but also axillary racemes of dichasial cymes, bisexual, protandrous; cyme 10–15 cm long, with 2 female flowers at apex and 7–9 male flowers at base, peduncle 2–5 cm long, glabrous; bracts deciduous, ovate to triangular, apex acuminate, margin entire, 0.7–1.5 cm long, 0.4–0.6 cm wide. Male flower: pedicel 2–2.8 cm long, glabrous; tepals 4, white to pinkish, outer 2 ovate or orbicular, 1.9–2.3 cm long, 1.5–2 cm wide, inner 2, broadly oblong, 1.8–2.3 cm long, 0.5–1.5 cm wide, glabrous; androecium actinomorphic, subglobose, ca. 0.7 cm across, stamens 60–80, yellow, clavate; filaments ca. 2 mm long, fused to a short central column; anthers 1–1.2 mm long, apex truncate. Female flower: pedicel 1.7–3.5 cm long, glabrous; ovary white, wings 3, manifestly unequal, 2 side wings almost undeveloped, abaxial wing much protruded, white to pale greenish or pinkish, 1-locular; placentation parietal, placentae 2, each bilamellate; tepals 5, white in the greenhouse (pinkish in the wild), unequal, elliptic to obovate, 1.5–2 cm long, 0.5–1.1 cm wide, apex obtuse; styles 2, ca. 5 mm long, 2- or 3-cleft, fused at base, stigmatic band wavy-twisted and spiralled. Capsule nodding, stalk 3.5–5.5 cm long, abaxial wing triangular to rectangular, 2.4–3.8 cm tall, 2.0–2.4 cm wide, lateral two wings barely developed, rounded, 0.2–0.4 cm tall, 1.8–2.2 cm wide. Seeds barrel-shaped, 0.25–0.3 mm long.

### Distribution and habit

Known only from the type locality.

### Etymology

The epithet refers to Myanmar (formerly Burma) where it was discovered.

### Additional specimens examined

MYANMAR. Sagaing Region: Alang Daw Kathapa National Park, 22°18′47.7″ N, 94°28′32.7″ E, 438 m, 2 Feb 2012, *Peng 23565* (HAST); 22°18′49.5″ N, 94°28′30.2″ E, 434 m, 2 Feb 2012, *MM*-*0556* (KB); 22°18′47.7″ N, 94°28′32.7″ E, 438 m, 2 Feb 2012, *MM*-*0611* (KB, HHU); 22°18′44.2″ N, 94°28′28.7″ E, 380 m, 2 Feb 2012, *MM*-*0616* (KB); 22°19′25″ N, 94°29′37.7″ E, 380 m, 5 Feb 2012, *MM*-*0848* (KB, RAF).

### Chromosome cytology

Somatic chromosome at metaphase of *B. myanmarica* were shown to be 2*n* = 38 in this study (Fig. 4c).

## Discussion

**Fig. 3:**
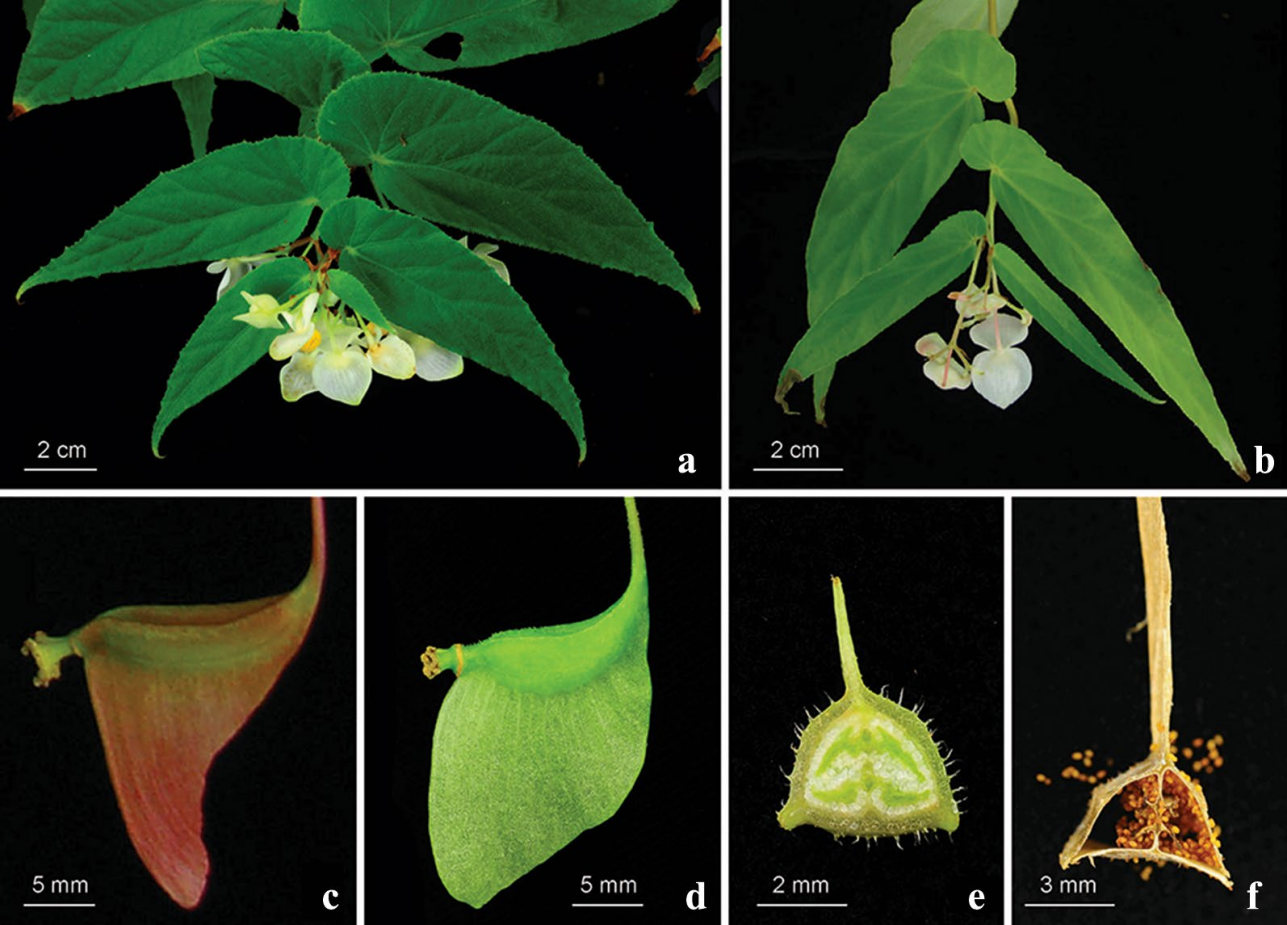
Overview of leaves, fruit and cross section of ovary in *Begonia griffithiana* and *B. nepalensis*. **a**, **b** Leaves. **c**, **d** Fruit. **e**, **f** Cross section of ovary. *Begonia griffithiana* (**a**, **c**, **e**). *Begonia nepalensis* (**b**, **d**, **f**)

**Fig. 4:**
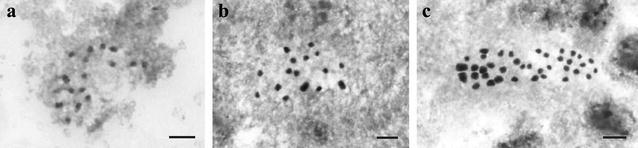
Somatic chromosomes at metaphase of *Begonia*. **a** *B. griffithiana* (2*n* = 16, *Peng 20851*). **b** *B. nepalensis* (2*n* = 16, *Peng 20854*). **c** *B. myanmarica* (2*n* = 38, *Peng 23566*). *Scale bar* 5 µm

**Fig. 5:**
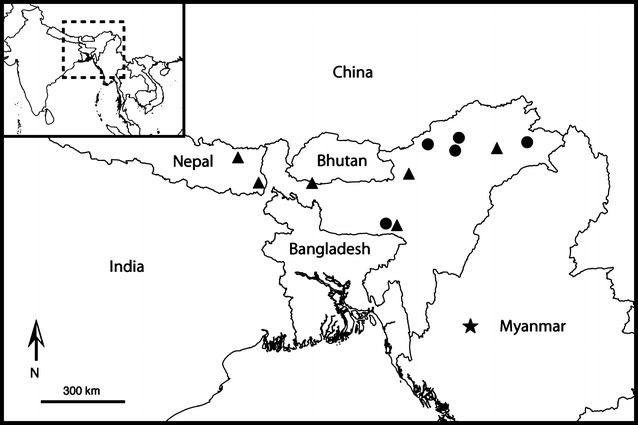
Distribution map of *Begonia griffithiana* (*circle*), *B. myanmarica* (*star*), *B. nepalensis* (*trangle*). Distribution data of *B. griffithiana* and *B. nepalensis* is based on GPS data in the Begonia Resource Center (Hughes et al. 2015)

*Begonia myanmarica* has only one developed wing in ovary/fruit (Figs. 2k, 3c, d), the key character of sect. *Monopteron* in *Begonia* (Doorenbos et al. 1998). The new species, however, deviates from sect. *Monopteron* with axillary placentation and two locules in ovary (Fig. 3e, f) in having 1-locular ovary and parietal placentation (Fig. 2j). Additionally, *B. myanmarica* has ovate to broadly ovate leaves and large leaves (ca. 20–40 cm long, 22–30 mm across) (Fig. 2b, c), whereas leaves of *B. griffithiana* and *B. nepalensis* are lanceolate to oblong and no longer than 20 × 10 cm (Fig. 3a, b). Cytologically, somatic chromosome of *B. myanmarica* is determined to be 2*n* = 38 (Fig. 4c), while chromosomes of *B. griffithiana* and *B. nepalensis* are both 2*n* = 16 (Fig. 4a, b) in our study. Geographically, *B. myanmarica* is endemic to Myanmar while *B. griffithiana* and *B. nepalensis* are distributed in India, Nepal and Bhutan (Fig. 5). We concluded that *B. myanmarica* is sharply distinct from *B. griffithiana* and *B. nepalensis.*

**Fig. 6:**
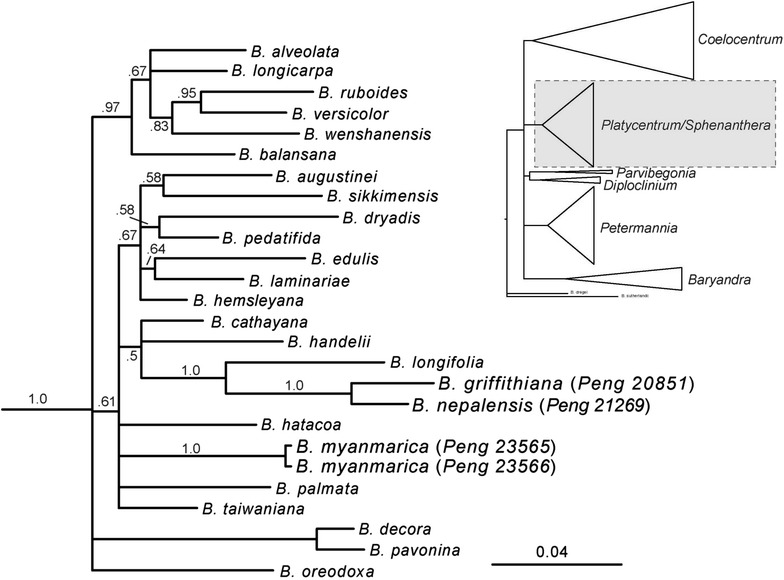
Phylogenetic tree of section *Platycentrum*-section *Sphenanthera* in *Begonia* generated from Bayesian analysis of nrITS sequence data. *Numbers on the branches* indicate posterior probability of Bayesian inference analysis. The *inset* is simplified phylogenetic tree based on the nrITS dataset and sectional classification of Chung et al. (2014)

In our molecular phylogenetic study, *B. griffithiana* and *B. nepalensis* form a strongly supported clade (posterior probability, PP = 1) nested within the clade dominated by sect. *Platycentrum*-sect. *Sphenanthera* clade [Fig. 6; Clade *PLA*-*SPH* in Chung et al. (2014)], congruent with the studies of Rubite (2010), Thomas (2010), Rajbhandary et al. (2011), and Leong (2017). Two sampled individuals of *B. myanmarica* also fall within the Clade *PLA*-*SPH* but not clustered within sect. *Monopteron* clade (Fig. 6), suggesting that the fruit morphology of a single developed wing in the ovary/fruit is homoplasious. Morphologically, the key characters for sect. *Platycentrum*-sect. *Sphenanthera* clade are evergreen, rhizomatous and two locules in ovary (Leong 2017). *Begonia myanmarica* is evergreen with stout rhizome, but having 1-locular ovary. Compared with other species in sect. *Platycentrum*-sect. *Sphenanthera*, *B. myanmarica* is unique with a single developed wing and having 1-locular ovary not known in any other taxa in this clade. Further studies with increasing sampling of Myanmar *Begonia* are needed to place *B. myanmarica* in its proper infrageneric position.

## Conclusion

Studies of morphology, molecular phylogenetics and cytology support the recognition of the new species, *Begonia myanmarica*, which is fully described and illustrated. Our results also indicate that *B. myanmarica* is not closely related to species previously assigned to sect. *Monopteron*, suggesting that the fruit morphology of a single developed wing in the ovary/fruit characterizing sect. *Monopteron* is homoplasious.

## Appendix

Voucher information and GenBank accession numbers in newly generated sequences and morphological observation are listed here. Voucher data is given using following format: Taxon name, Collection locality, collector(s) and collector number (herbarium for voucher specimen), GenBank accession numbers for nrITS. Other nrITS sequences refer to Chung et al. (2014).

### Newly generated sequences

*Begonia myanmarica* C.I Peng & Y.D. Kim, Myanmar, *Peng 23565* (HAST), KY088184; *Peng 23566* (HAST), KY088185. *Begonia griffithiana* Warb., India, *Peng 20851* (HAST), KY088186. *Begonia nepalensis* Warb., India, *Peng 20854* (HAST), KY088187.

### Other sequences from Chung et al. (2014)

*Begonia aequata* A. Gray, AF485147; *Begonia alicida* C.B.Clarke ex Hook. f., KF636419; *Begonia alveolata* Yu, AY048977, *Begonia amphioxus* Sands, AF485150; *Begonia arachnoidea* C.I Peng, Yan Liu & S.M. Ku, KF636420; *Begonia augustinei* Hemsl., KF636421; *Begonia auritistipula* Y.M. Shui & W.H. Chen, KF636422; *Begonia austroguangxiensis* Y.M. Shui & W.H. Chen, KF636423; *Begonia balansana* Gagnep., AF485091; *Begonia bamaensis* Yan Liu & C.I Peng, KF636424; *Begonia bataiensis* Kiew, KF636425; *Begonia berhamanii* Kiew, KF636426; *Begonia bipinnatifida* J.J.Sm., KF636427; *Begonia boisiana* Gagnep., AF534719; *Begonia bolsteri* Merr., KF636428; *Begonia brevipes* Merr., KF636429; *Begonia brevirimosa* Irmsch., AF485145; *Begonia cathayana* Hemsl., AF280106; *Begonia cavaleriei* H.Lév., KF636430; *Begonia chingii* Irmsch., KF636432; *Begonia cirrosa* L.B.Sm. & Wassh., AY048979; *Begonia contracta* Warb., KF636433; *Begonia cylindrica* D.R.Liang & X.X.Chen, KF636434; *Begonia decora* Stapf, KF636435; *Begonia dipetala* Graham, AF469124; *Begonia dregei* Otto & A.Dietr., AY429336; *Begonia dryadis* Irmsch., KF636436; *Begonia edulis* H.Lév., KF636437; *Begonia erythrogyna* Sands, KF636438; *Begonia fimbristipula* Hance, KF636439; *Begonia fuscisetosa* Sands, KF636440; *Begonia goegoensis* N.E.Br., AF485138; *Begonia grandis* subsp. *holostyla* Irmsch., AF485088; *Begonia griffithiana* Warb., KY088186; *Begonia gueritziana* Gibbs, KF636441; *Begonia guixiensis* Yan Liu, S.M. Ku, C.I Peng, KF636442; *Begonia hainanensis* Chun & F.Chun, KF636443; *Begonia handelii* Irmsch., AY048982; *Begonia hatacoa* Buch.-Ham. ex D. Don, KF636444; *Begonia hemsleyana* Hook. f., AF485099; *Begonia hernandioides* Merr., KF636445; *Begonia inostegia* Stapf, KF636446; *Begonia isoptera* Dryand. ex Sm., KF636447; *Begonia jingxiensis* D. Fang & Y.G. Wei, KF636448; *Begonia kinabaluensis* Sands, KF636450; *Begonia kingiana* Irmsch., KF636451; *Begonia labordei* H.Lév., KF636452; *Begonia lagunensis* Elmer, KF636453; *Begonia lambii* Kiew, KF636454; *Begonia laminariae* Irmsch., KF636455; *Begonia lanternaria* Irmsch., KF636456; *Begonia leprosa* Hance, KF636457; *Begonia liuyanii* C.I Peng, S.M. Ku & W.C. Leong, KF636458; *Begonia longa* C.I Peng & W.C. Leong, KF636459; *Begonia longicarpa* K.Y. Guan & D.K. Tian, AY048985; *Begonia longifolia* Blume, AF485105; *Begonia longistyla* Y.M.Shui & W.H.Chen, KF636460; *Begonia luzhaiensis* T.C. Ku, KF636461; *Begonia madaiensis* Kiew, KF636462; *Begonia masoniana* Irmsch. ex Ziesenh., KF636463; *Begonia merrittii* Merr., KF636464; *Begonia nigritarum* Steud., KF636465; *Begonia ningmingensis* D. Fang, Y.G. Wei & C.I Peng, KF636466; *Begonia oreodoxa* Chun & F.Chun ex C.Y.Wu & T.C.Ku, KF636467; *Begonia oxysperma* A.DC., AF485131; *Begonia palmata* D.Don, KF636468; *Begonia panayensis* Merr., KF636469; *Begonia paracauliflora* sp. ined., KF636470; *Begonia parvula* H. Lév. & Vaniot, KF636471; *Begonia pavonina* Ridl., KF636472; *Begonia pedatifida* H.Lév., KF636473; *Begonia peltatifolia* H.L. Li, KF636474; *Begonia pengii* S.M. Ku & Yan Liu, KF636475; *Begonia pseudolateralis* Warb., KF636476; *Begonia pulvinifera* C.I Peng & Yan Liu, KF636477; *Begonia ramosii* Merr., KF636478; *Begonia ravenii* C.I Peng & Y.K.Chen, KF636479; *Begonia retinervia* D.Fang, D.H.Qin & C.I Peng, KF636480; *Begonia ruboides* C.M. Hu ex C.Y.Wu & T.C.Ku, KF636481; *Begonia rufipila* Merr., KF636482; *Begonia semiparietalis* Yan Liu, S.M.Ku & C.I Peng, KF636483; *Begonia serratipetala* Irmsch., KF636484; *Begonia sikkimensis* A.DC., KF636485; *Begonia sinofloribunda* Dorr, KF636486; *Begonia subnummularifolia* Merr., KF636487; *Begonia sutherlandii* Hook. f., AF485215; *Begonia symsanguinea* L.L. Forrest & Hollingsw., AF485151; *Begonia taiwaniana* Hayata, KF636488; *Begonia variabilis* Ridl., AY753732; *Begonia variegata* Y.M. Shui & W.H.Chen, KF636489; *Begonia versicolor* Irmsch., AF485090; *Begonia wadei* Merr. & Quisumb., KF636490; *Begonia wenshanensis* C.M. Hu ex C.Y. Wu & T.C. Ku, AY048974; *Begonia yappii* Ridl., KF636491.

### Morphological observation

*Begonia myanmarica* C.I Peng & Y.D. Kim, Myanmar, *Peng 23565* (HAST); *Peng 23566* (HAST). *Begonia griffithiana* Warb., India, *Peng 20851* (HAST). *Begonia nepalensis* Warb., India, *Peng 20854* (HAST).

*Begonia cathayana* Hemsl., China, *Peng 20288* (HAST). *Begonia decora* Stapf, Malaysia, *Peng 20261* (HAST). *Begonia dryadis* Irmsch., China, *Peng 18016* (HAST). *Begonia edulis* H.Lév., China, *Peng 18747* (HAST). *Begonia hatacoa* Buch.-Ham. ex D. Don, *Peng 20861* (HAST). *Begonia hemsleyana* Hook. f., China, *Peng 17590* (HAST). *Begonia laminariae* Irmsch., China, *Peng 17447* (HAST). *Begonia oreodoxa* Chun & F. Chun ex C.Y. Wu & T.C. Ku, China, *Peng 20454* (HAST). *Begonia palmata* D. Don, Taiwan, *Peng 20993* (HAST). *Begonia pavonina* Ridl., Malaysia, *Peng 20239* (HAST). *Begonia pedatifida* H. Lév., China, *Peng 18779* (HAST). *Begonia sikkimensis* A. DC., China, *Peng 20848* (HAST). *Begonia augustinei* Hemsl., China, *Peng 20759* (HAST). *Begonia versicolor* Irmsch., China, *Peng 18688* (HAST). *Begonia balansana* Gagnep., Vietnam, *Peng 21928* (HAST). *Begonia handelii* Irmsch., China, *Peng 17513* (HAST). *Begonia longifolia* Blume, Taiwan, *Peng 16795* (HAST).
